# Scholarly Communication and Authorship Pattern of Indian Institute of Management Ahmedabad: a Scientometric Analysis

**DOI:** 10.12688/f1000research.188052.1

**Published:** 2026-08-20

**Authors:** Sandeep Kumar Mohanty, Dr. Dillip K Swain, Chandrakanta Swain, Bijayalaxmi Rautaray

**Affiliations:** 1 Library and Information Science, Kalinga Institute of Industrial Technology, Bhubaneswar, Odisha, 751024, India; 2 Library and Information Science, Kalinga Institute of Industrial Technology, Bhubaneswar, Odisha, 751024, India; 3 Library, Indian Institute of Management Raipur, Raipur, Chhattisgarh, India; 4 Library and Information Science, Kalinga Institute of Industrial Technology, Bhubaneswar, Odisha, 751024, India

**Keywords:** Authorship Pattern, Lotka's Law, Degree of Collaboration, VOSviewer, Co-Occurance Keyword, IIMA

## Abstract

**Background:**

Management research has grown substantially over the past few decades, driven by globalization, digital transformation, sustainability challenges, and technological innovation. These developments have also transformed scholarly communication, with collaborative and interdisciplinary research increasingly replacing single-author publications. Scientometric analysis provides a systematic approach to evaluating research productivity, collaboration patterns, and scholarly impact.

**Method:**

The study employs a scientometric approach using 2,981 Scopus-indexed publications of the Indian Institute of Management Ahmedabad (1967–2025). The data were analyzed using bibliometric indicators, including publication growth, authorship patterns, degree of collaboration, author productivity (Lotka's Law), and keyword co-occurrence analysis through VOSviewer.

**Results:**

The findings reveal a substantial increase in research productivity, particularly during the last decade, indicating the institute's growing research capacity and global academic visibility. Collaborative research dominates the publication output, with a Degree of Collaboration of 0.75, reflecting the increasing importance of interdisciplinary and multi-authored research. The author productivity pattern does not conform to the classical Lotka's inverse-square law; instead, the estimated Lotka exponent (n ≈ 2.88) indicates a steeper decline in author productivity.

**Conclusion:**

The study demonstrates the Indian Institute of Management Ahmedabad’s evolution into a highly productive, collaborative, and internationally recognized research institution.

## Introduction

Scholarly communication, or communication between and among researchers and other experts in a given field, may be considered *primary source of information* if it is original communication as the trends of research or research productivity ( Welsh & Wright, 2010; Aziz, F., et al 2024). Research productivity is widely recognized as an important indicator of the academic performance and scholarly impact of higher education institutions. Scientometric studies provide a systematic approach to assessing research output by analyzing publication trends, authorship patterns, and author productivity ( Davarpanah &Aslekia, 2008). Such analyses help identify the growth of research, patterns of collaboration, and the distribution of scholarly contributions within a specific institution or discipline.

Management is a multidisciplinary field encompassing areas such as strategic management, human resource management, organizational behavior, marketing, finance, operations management, entrepreneurship, and innovation. The increasing volume of scholarly publications has made bibliometric analysis an effective method for understanding research productivity, collaboration, and emerging themes. Research trends help identify the evolution of knowledge, while authorship patterns reveal the nature of scientific collaboration, institutional partnerships, and international research networks. In this regard, the Indian Institute of Management Ahmedabad (IIMA), one of India's leading management institutions, has made substantial contributions to management research through its faculty and research community. Evaluating the institute's publication output offers valuable insights into the evolution of its research activities and the characteristics of its scholarly contributions. In this context, the present study examines the research trends and authorship pattern of publications produced by IIMA. It analyzes the year-wise growth of publications from 1967 to 2025, investigates authorship patterns, and evaluates author productivity using Lotka's Law to determine the extent to which the observed productivity distribution conforms to the theoretical model.

The findings reveal the institute's contribution to research over the years and highlight its changing publication patterns, indicating the growth and development of its research activities. Moreover, the analysis provides insights into the productivity of authors, the concentration of scholarly contributions, and the overall characteristics of research output at the institute.

Contextually, the study explores to find answers to the following research questions:

1. What is the year-wise distribution of research publications produced by the Indian Institute of Management Ahmedabad (IIMA) during the period 1967–2025?
2. What are the trends and growth patterns in the research output of IIMA over the study period?
3. What are the authorship patterns and levels of author productivity in the publications produced by IIMA?
4. To what extent does the observed distribution of author productivity in IIMA publications conform to Lotka's Law of scientific productivity?

## Literature review

Earlier bibliometric studies have extensively examined research productivity, publication trends, authorship patterns, and collaboration across academic institutions and disciplines. Gopikuttan and Aswathi (2014) analyzed the research productivity of the University of Kerala's science departments during 2000–2012 using the Web of Science database. The study identified 1,068 publications and reported a steady increase in research output, with Biology, Chemistry, and Engineering emerging as the most productive disciplines. Collaborative research dominated the authorship pattern, while Dr. T. S. Anirudhan was identified as the most productive and highly cited author. Similarly, Rajgoli and Laxminarsaiah (2015) examined 1,487 articles published in the *Journal of Spacecraft and Rockets* (2001–2011) and found that multi-authored papers constituted 87.15% of the total publications. The study also reported that author productivity only partially conformed to Lotka's Law.

Sahoo et al. (2017) investigated research productivity in Indian management institutions and emphasized that publication count, citations, h-index, and journal rankings are important indicators of research performance. The study highlighted that institutional culture, doctoral training, and research-oriented policies significantly influence research productivity and noted a gradual shift among Indian management institutions from teaching-oriented to research-intensive environments. Behl et al. (2020) discussed the growing importance of the "publish or perish" culture in higher education. The authors observed that while publication incentives have increased research productivity, they have also intensified publication pressure and raised concerns regarding research quality and unethical publishing practices. The study further emphasized that international collaboration enhances research visibility and citation impact, although Indian business management research continues to lag in global collaborative networks.

Torres and Manjarrez-Dominguez (2021) examined Open Access (OA) publishing practices among accounting faculty and found that OA adoption remains relatively limited despite its advantages in improving research visibility and citation impact. Their bibliometric analysis demonstrated a gradual increase in OA publications between 2013 and 2018, with institutions publishing more OA articles receiving higher citation impact.

Doulani (2021) reported that both the scientific output of Alzahra University, measured by the number of publications, and the citations received by these publications increased steadily over the study period. At the national level, the university's most significant scientific collaboration was with researchers from the University of Tehran.

Singh et al. (2023) conducted a bibliometric analysis of the Indian Institutes of Management (IIMs) for the period 2010–2019 using the Scopus database. The study examined publication trends, citation performance, thematic areas, international collaboration, gender distribution, and open-access publishing. The findings revealed that first-generation IIMs, particularly IIM Ahmedabad, produced the highest research output, while newer institutions such as IIM Udaipur exhibited stronger international collaboration. The authors concluded that Indian management institutions need to improve research quality and strengthen international partnerships to achieve greater global recognition.

Tyagi (2024) carried out a comprehensive bibliometric study of the top four Indian Institutes of Management during 2010–2021 using Scopus data. The analysis identified 4,397 publications and demonstrated a consistent increase in research productivity. IIM Ahmedabad emerged as the leading institution in publication output, while collaboration extended to more than 125 countries, particularly the United States and the United Kingdom. The study also examined citation impact, authorship patterns, and core journals, highlighting the significant contribution of these institutions to global management research. Duan (2024) analyzed 1,792 publications on FinTech research indexed in the Web of Science between 1999 and 2022. The study reported rapid growth in publication output, although collaboration among individual authors remained relatively limited. In contrast, international collaboration was extensive, involving researchers from 77 countries. The findings also emphasized the interdisciplinary nature of FinTech research, with increasing integration of blockchain, artificial intelligence, and big data technologies. Asif & Fazel (2024) revealed a significant growth in annual scientific production on digital technology and tourism, peaking at 100 articles in 2023 and China was the leader in research output, and “Current Issues in Tourism” and “Information Technology and Tourism” were found to be the influential journals. Rostami et al. (2024) reviewed research productivity across several universities and consistently reported substantial growth in publication output, particularly after 2005. Their findings demonstrated that international collaboration significantly enhanced research quality, citation impact, and institutional visibility. The study also highlighted chemistry, physics, and medical sciences as the most productive research areas across the institutions examined.

Islam et al. (2025) conducted a bibliometric analysis of big data research in librarianship and demonstrated a substantial increase in both publications and citations, indicating the growing scholarly interest in this emerging field. The study identified China and the United States as the leading contributors to research output, reflecting their strong investment in big data technologies and information science. Singh (2025) in his study on modelling analysis of Virtual Reference Services (VRS) research published between 2000 and 2023 using the Scopus database. Based on 1,632 publications, the study identified a sharp increase in research activity following the COVID-19 pandemic. The findings indicated that while research output has expanded considerably, collaboration among authors and institutions remains fragmented. Emerging research themes included artificial intelligence, chatbots, and augmented reality, reflecting the technological transformation of library services.

Kashani and Dastani (2025) conducted a comprehensive bibliometric analysis of 41,352 scientific publications on artificial intelligence (AI) in the medical sciences to evaluate publication trends and map the intellectual structure of the field through keyword co-occurrence analysis. The study revealed a rapid expansion of AI-related medical research and identified major thematic clusters, highlighting the evolving research priorities and emerging areas of interest. Maheshwari (2025) examined the role of responsible leadership in advancing corporate social responsibility (CSR) and enhancing organizational performance. The study demonstrated that transformational, ethical, and responsible leadership styles positively influence CSR initiatives and stakeholder engagement, with ethical organizational culture and intellectual capital acting as important mediating mechanisms.

Although previous studies have extensively explored publication trends, research productivity, collaboration patterns, and citation impact across universities and management institutions, limited attention has been given to a comprehensive scientometric analysis focusing exclusively on the Indian Institute of Management Ahmedabad (IIMA). Furthermore, studies examining author productivity through the application of Lotka's Law in the context of management research remain scarce. Therefore, the present study addresses this gap by analyzing the year-wise publication trends, authorship patterns, and author productivity of IIMA's research output from 1967 to 2025, while evaluating the applicability of Lotka's Law to its publication data.

## Objectives of the study

The following are the key objectives of the study:

1. To analyze the year-wise distribution and growth pattern of publications produced by the Indian Institute of Management Ahmedabad (IIMA) during the study period (1967–2025);
2. To examine the authorship pattern and degree of collaboration among researchers of IIMA;
3. To evaluate the author productivity pattern of IIMA publications using Lotka’s Law and determine the conformity of the observed productivity distribution;
4. To identify the most productive authors and assess their contribution to the overall research output of the institute; and
5. To study the publication trends of IIMA through scientometric indicators, including publication output and collaborative research patterns.

## Methodology

The present study adopts a quantitative bibliometric approach to examine the research output of the Indian Institute of Management Ahmedabad (IIMA **)**. Bibliographic data were retrieved from the Scopus database, yielding a total of 2,981 publications published during the period 1967–2025 **.** The year 2026 was excluded from the analysis because the publication data for the year were incomplete at the time of data collection. The retrieved records were exported and processed for bibliometric analysis. Descriptive indicators such as annual publication output, authorship pattern, and collaborative research trends were analyzed. The Degree of Collaboration (DC) was computed using the formula proposed by Subramanyam (1983) to determine the extent of collaborative research. The authorship productivity pattern was examined using Lotka's Law **,** and the goodness-of-fit of the observed distribution was evaluated by estimating the modified Lotka exponent (n) using the logarithmic least-squares method.

To visualize the intellectual and collaborative structure of IIMA's research output, VOSviewer (version X.X) was employed to construct author keyword co-occurrence visualization maps. The collected data were organized, cleaned, and analyzed using appropriate bibliometric techniques to ensure the accuracy and reliability of the findings.

## Findings and results

The results of the bibliometric analysis are presented in accordance with the objectives and methodology of the study. The analysis examines the publication output of the Indian Institute of Management Ahmedabad (IIMA) from 1967 to 2025, focusing on annual research productivity, authorship patterns, and collaborative research trends. Bibliometric indicators, including the Degree of Collaboration (DC) used to evaluate the extent and nature of research collaboration. The applicability of Lotka's Law to the observed authorship productivity is also assessed using the modified Lotka exponent. In addition, author keyword co-occurrence maps generated using VOSviewer are presented to illustrate the major research themes and the intellectual structure of IIMA's scholarly output. The findings are discussed under with appropriate tables and figures with interpretations.

Table 1 shows a total of 2,981 documents that are analyzed for their year-wise distribution. The year-wise distribution of documents indicates a clear increase in research output in recent years. Among the individual years, 2022 recorded the highest number of publications with 214 articles (7.18%) **,** followed by 2021 with 181 articles (6.07%) **,** 2019 with 179 articles (6.00%) **,** and 2023 with 177 articles (5.94%) **.** The publication trend remained consistently high from 2019 to 2025 **,** with each year contributing approximately 7% of the total documents, reflecting sustained research activity during this period.

**Table 1.** Year-wise distribution of documents.

Sl No	Year	No of articles	Cummulative articles	% out of 2981	Cummulative %
1	2025	158	158	5.30	5.30
2	2024	155	313	5.20	10.50
3	2023	177	490	5.94	16.44
4	2022	214	704	7.18	23.62
5	2021	181	885	6.07	29.69
6	2020	169	1054	5.67	35.36
7	2019	179	1233	6.00	41.36
8	2018	146	1379	4.90	46.26
9	2017	156	1535	5.23	51.49
10	2016	129	1664	4.33	55.82
11	2015	90	1754	3.02	58.84
12	2014	65	1819	2.18	61.02
13	2013	68	1887	2.28	63.30
14	2012	68	1955	2.28	65.58
15	2011	71	2026	2.38	67.96
16	2010	55	2081	1.84	69.80
17	1967 to 2009	900	2981	30.19	100

A moderate level of publication was observed during 2016–2018 **,** with annual contributions ranging from 4.33% to 5.23% **.** In contrast, the years 2010–2015 showed comparatively lower research output, with annual contributions between 1.84% and 3.02% **.** The 1967–2009 period collectively accounted for 900 articles (30.19%) representing the largest proportion because it combines publications spanning more than four decades rather than a single year.

Overall, the distribution suggests that research productivity has increased over time, with a noticeable concentration of publications in the last decade. Figure 1 reflects growing scholarly interest and expanding research activity in the field, particularly after 2018.

**Figure 1.**
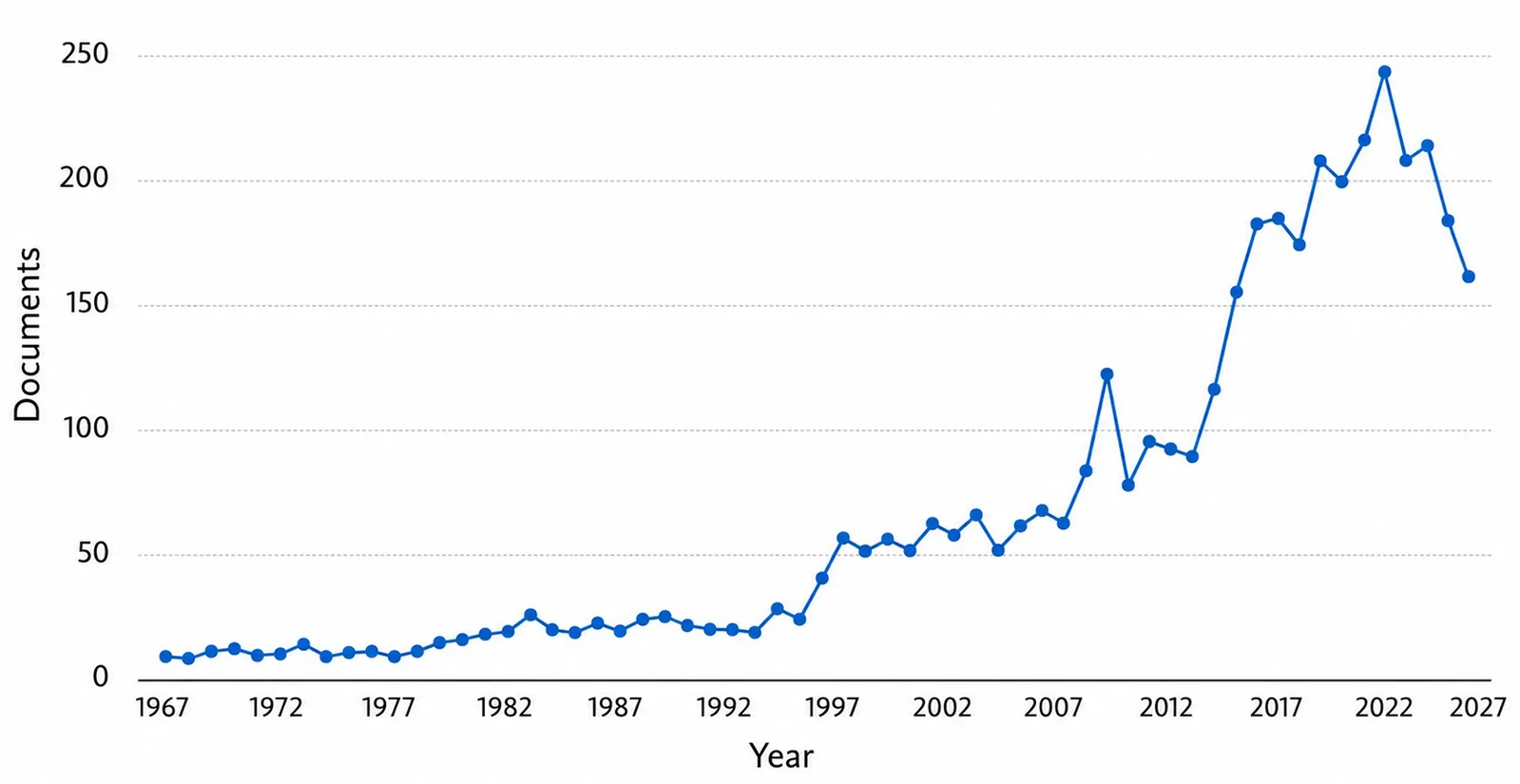
Year-wise distribution of documents.

Table 2 reveals that Garg, A **.** is the leading contributor with 80 publications (2.68%) **,** followed closely by Shukla, P. R. with 79 publications (2.65%) and Singh, S. with 75 publications (2.52%) **.** Roy, D. (70; 2.35% **)** and Noronha, E. (65; 2.18%) also rank among the top contributors. The remaining authors **—**Vohra, N. **,** Chakrabarti, A. S. **,** Sinha, A. **,** Laha, A. K. **,** and Dutta, G. each contributed between 1.21% and 1.71% of the total 2,981 publications. Although each individual author's share is relatively small, these researchers have made consistent and significant contributions to the field, indicating that the research output is distributed across a broad group of active scholars rather than being dominated by a single author which is further demonstrated in Figure 2.

**Table 2.** Prolific contributors.

Sl No	Author	No of articles	% out of 2981
1	Garg, A	80	2.68
2	Shukla, P R	79	2.65
3	Singh, S	75	2.52
4	Roy, D	70	2.35
5	Noronha, E	65	2.18
6	Vorha, N	51	2.71
7	Chakrabarti, A S	49	1.64
8	Sinha, A	48	1.61
9	Laha, A K	41	1.38
10	Dutta, G	36	1.21

**Figure 2.**
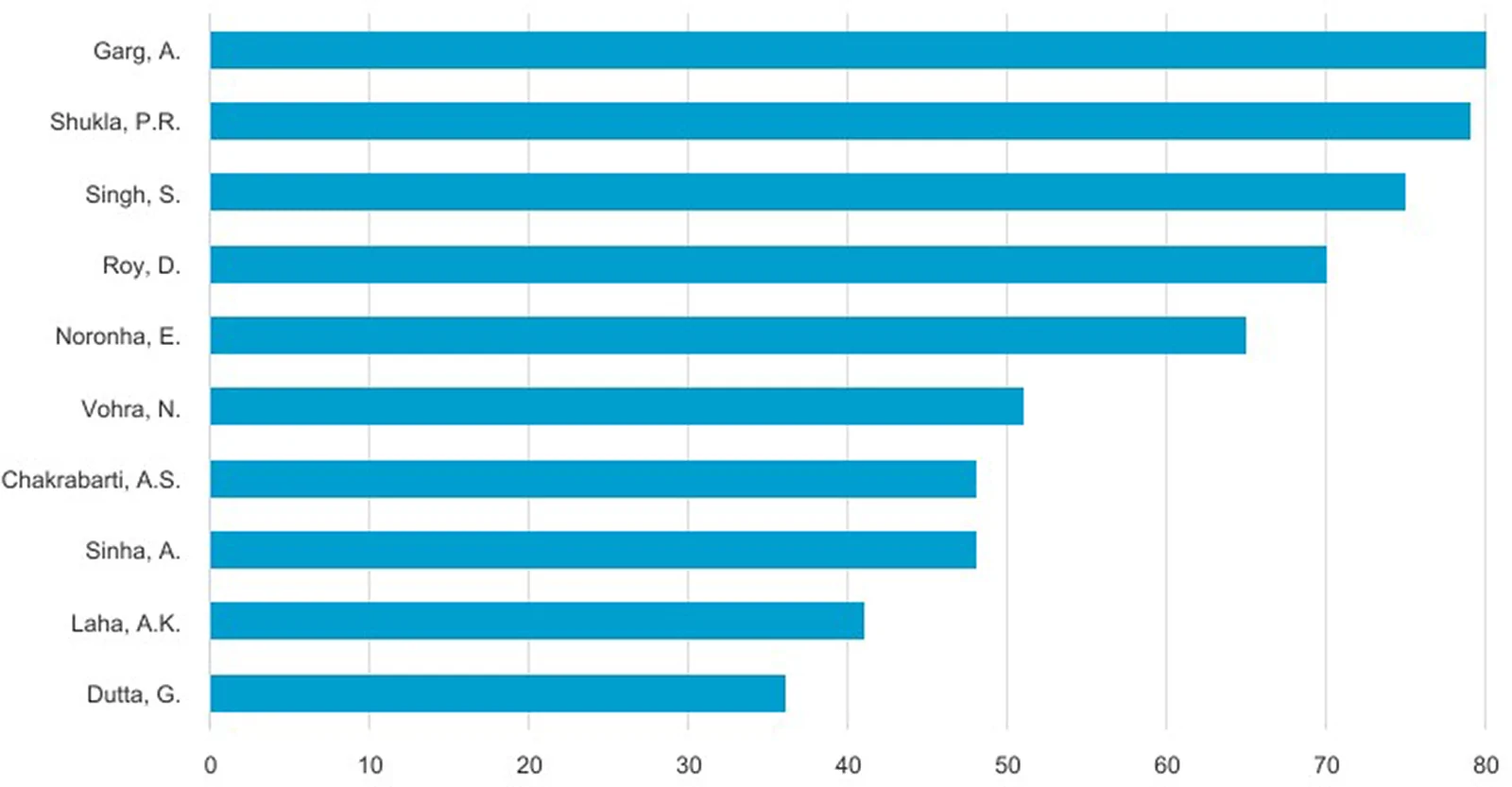
Prolific contributors of IIM, Ahamedabad.

It is evident from Table 3 that the top 10 most-cited publications demonstrate significant research impact across diverse disciplines, including artificial intelligence, organizational behavior, statistics, public health, optimization, and supply chain management. Most importantly, all are collaborative papers. The papers are published in prestigious international journals such as *Science*, *The Lancet*, *IEEE Transactions on Evolutionary Computation*, and the *International Journal of Information Management*, reflecting high research quality and global visibility.

**Table 3.** Highly Cited top 10 Papers.

Sl No	Title of the paper	Author(s)	Name of the journal	Year of publication	Scopus Citations
1	“So what if ChatGPT wrote it?” Multidisciplinary perspectives on opportunities, challenges and implications of generative conversational AI for research, practice and policy	Dwivedi, et al	International Journal of Information Management	2023	3908
2	Differences between tight and loose cultures: A 33-nation study	Gelfand et al	Science	2011	2524
3	A network algorithm for performing fisher’s exact test in r × c contingency tables	• Mehta and Patel	Journal of the American Statistical Association	1983	1071
4	Global, regional, and national prevalence of adult overweight and obesity, 1990–2021, with forecasts to 2050: a forecasting study for the Global Burden of Disease Study 2021	Ng, Marie et al	The Lancet	2025	888
5	Culture specific and crossculturally generalizable implicit leadership theories: Are attributes of charismatic/transformational leadership universally endorsed?	Den Hartog et al	Leadership Quarterly	1999	856
6	A Review on Bilevel Optimization: From Classical to Evolutionary Approaches and Applications	Sinha A.,Malo P.,Deb K.	IEEE Transactions on Evolutionary Computation	2018	850
7	Culture, Emotion Regulation, and Adjustment	Matsumoto et al	Journal of Personality and Social Psychology	2008	813
8	Robotized and automated warehouse systems: Review and recent developments	Azadeh K.,De Koster R.,Roy D.	Transportation Science	2019	512
9	Covid-19′s impact on supply chain decisions: Strategic insights from NASDAQ 100 firms using Twitter data	Sharma, Adhikary and Borah	Journal of Business Research	2020	433
10	Computing an exact confidence interval for the common odds ratio in several 2×2 contingency tables	Mehta, Patel and Gray	Journal of the American Statistical Association	1985	412

*Note*: Citation data was retrieved on 8 July, 2026

The exceptionally high citation counts indicate that these publications have made substantial theoretical and practical contributions. Foundational methodological papers (e.g., Mehta & Patel) continue to receive citations over several decades, demonstrating their enduring relevance, while recent publications on ChatGPT, COVID-19, and obesity highlight the researchers' responsiveness to emerging global challenges. Additionally, several review and conceptual papers have achieved high citation impact by synthesizing existing knowledge and guiding future research.

Based on the summarized authorship data obtained from Table 4 and presented in Table 5, it is found that the authorship pattern of the 2,981 publications reveals a strong preference for collaborative research over individual contributions. Of the total publications, 732 (24.55%) were written by a single author **,** whereas 2,249 (75.45%) were produced through collaborative efforts involving two or more authors. This indicates that collaboration has become the dominant mode of research in the field.

**Table 4.** Year-wise distribution of Authorship pattern.

Year	Single Author	Two Author	Three Authors	Four Authors	Five Authors	Six Authors	Seven Authors	Eight Authors	Nine Authors	Ten Authors	More than Ten Authors	Total
1967	1	0	0	0	0	1	0	0	0	0	0	2
1968	1	1							0	0	0	2
1969	3	1	0	0	0	0	0	0	0	0	0	4
1970	2	1	1	0	0	0	0	0	0	0	0	4
1971	1	1	0	0	0	0	0	0	0	0	0	2
1972	1	1	0	0	0	0	0	0	0	0	0	2
1973	2	3	1	0	0	0	0	0	0	0	0	6
1974	2	1	0	0	0	0	0	0	0	0	0	3
1975	1	1	1	0	0	0	0	0	0	0	0	3
1976	3	1	0	0	0	0	0	0	0	0	0	4
1977	2	0	0	0	0	0	0	0	0	0	0	2
1978	2	1	1	0	0	0	0	0	0	0	0	4
1979	1	2	1	1	1	0	0	0	0	0	0	6
1980	5	1	0	0	0	0	0	0	0	0	0	6
1981	5	1	2	0	0	0	0	0	0	0	0	8
1982	4	4	0	0	0	0	0	0	0	0	0	8
1983	7	6	0	0	0	0	0	0	0	0	0	13
1984	4	3	1	0	0	0	0	0	0	0	0	8
1985	3	3	1	0	0	0	0	0	0	0	0	7
1986	2	6	1	0	0	0	0	0	0	0	0	9
1987	2	3	2	0	0	0	0	0	0	0	0	7
1988	3	5	2	0	0	0	0	0	0	0	0	10
1989	5	3	3	0	0	0	0	0	0	0	0	11
1990	7	0	2	0	0	0	0	0	0	0	0	9
1991	4	1	2	0	0	0	0	0	0	0	0	7
1992	3	2	1	0	1	0	0	0	0	0	0	7
1993	5	1	0	0	0	0	0	0	0	0	0	6
1994	7	4	2	1	0	0	0	0	0	0	0	14
1995	7	1	1	0	0	0	0	0	0	0	0	9
1996	14	8	1	0	0	0	0	0	0	0	0	23
1997	18	12	5	0	1	0	0	0	0	0	0	36
1998	18	9	1	2	0	0	0	0	0	1	0	31
1999	17	14	2	1	0	0	0	0	0	0	1	35
2000	17	10	4	0	0	1	0	0	0	0	0	32
2001	18	12	6	3	3	0	0	0	0	0	0	42
2002	16	10	4	6	1	0	0	0	0	0	0	37
2003	13	16	8	2	4	1	0	0	0	0	1	45
2004	14	8	3	3	0	0	0	1	1	1	1	32
2005	16	15	5	0	0	1	1	0	1	0	1	40
2006	14	13	9	5	1	1	0	1	0	1	1	46
2007	12	16	6	5	0	0	0	0	0	1	1	41
2008	24	17	7	5	2	1	1	1	0	0	2	60
2009	24	40	10	13	2	3	1	2	0	1	1	97
2010	16	25	9	1	0	2	0	1	1	0	0	55
2011	15	31	12	4	3	0	1	0	0	1	4	71
2012	25	23	10	4	3	0	0	0	1	1	1	68
2013	13	26	16	3	3	0	2	0	0	0	2	65
2014	24	31	19	8	0	3	1	1	1	0	2	90
2015	28	47	29	13	5	2	1	0	0	0	4	129
2016	31	60	43	9	7	1	1	1	0	0	3	156
2017	35	51	37	19	5	2	1	1	1	3	1	156
2018	35	42	39	16	6	3	2	0	1	1	1	146
2019	42	58	41	24	7	0	2	0	0	1	4	179
2020	24	50	42	33	4	2	1	1	1	2	9	169
2021	26	56	58	26	6	3	1	1	1	1	2	181
2022	35	48	63	43	8	6	1	1	1	2	6	214
2023	16	63	41	30	15	3	1	1	1	1	5	177
2024	19	38	65	35	10	5	3	1	1	3	6	186
2025	18	49	36	30	8	4	2	1	0	1	10	159
Total	732	956	656	345	106	45	23	15	12	22	69	2981

**Table 5.** Absract of Authorship pattern.

Authorship Pattern	Publications	Percentage (%)
Single Author	732	24.55
Two Authors	956	32.07
Three Authors	656	22.01
Four Authors	345	11.57
Five Authors	106	3.56
Six Authors	45	1.51
Seven Authors	23	0.77
Eight Authors	15	0.50
Nine Authors	12	0.40
Ten Authors	22	0.74
More than Ten Authors	69	2.31
Total	2,981	100.00

Among the different authorship categories, two-author publications constitute the largest share with 956 papers (32.07%) **,** followed by three-author publications with 656 papers (22.01%) **.** Together, these two categories account for 54.08% of the total publications, demonstrating that small collaborative teams are the most common form of scholarly output.

Publications involving four authors represent 345 papers (11.57%) **,** while those with five authors account for 106 papers (3.56%) **.** Papers with six, seven, eight, nine, and ten authors contribute relatively smaller proportions, accounting for 1.51% **,** 0.77% **,** 0.50% **,** 0.40%, and 0.74% **,** respectively. Notably, 69 publications (2.31%) involved more than ten authors, reflecting the emergence of large-scale collaborative and interdisciplinary research projects.

Overall, the findings indicate a clear shift from individual authorship toward collaborative research. The predominance of two- and three-author publications suggests that researchers increasingly prefer working in small teams, while the presence of publications with large numbers of authors highlights the growing importance of interdisciplinary collaboration, institutional partnerships, and international research networks. These trends are consistent with the evolving nature of modern scientific research, where collaboration enhances knowledge sharing, resource utilization, and research impact.

The Degree of Collaboration (DC) can be calculated using the formula proposed by K. Subramanyam (1983):

$$\mathrm{DC}=N_{m}/\left(N_{m}+N_{s}\right)$$

Where:

• N _m_= Number of multi-authored publications
• N _s_= Number of single-authored publications

### Calculation

• Single-authored publications (N _s_)= 732
• Multi-authored publications (N _m_) = 2,981 − 732 = 2,249

Degree of Collaboration (DC) = 2249/2981=0.75

The Degree of Collaboration (DC) is 0.75, indicating that 75.44% of the publications were produced through collaborative authorship. This reflects a high level of research collaboration, with three out of every four publications involving two or more authors. Such a value suggests that collaborative research is the predominant mode of scholarly communication in the dataset, highlighting the increasing importance of teamwork, interdisciplinary research, and institutional collaboration.

The observed author productivity pattern was compared with the expected distribution under the classical Lotka's Law (n = 2). Table 6 reveals a noticeable differences between the observed and expected frequencies, particularly among authors with more than ten publications. These deviations indicate that the observed distribution does not adequately fit the theoretical inverse-square distribution. Therefore, it is concluded that the author productivity pattern in the dataset does not conform to the classical Lotka's Law. Modified Lotka distribution can be further explored through the following formula:

Log(y)=log(C)−nlog(x)

**Table 6.** Authorship productivity pattern.

No of articles x	No of authors observed	Observed %	No of authors expected	Expected %
1	5398	63.22%	5,191	60.79
2	1383	16.20%	1,298	15.20
3	690	8.08%	577	6.75
4	424	4.97%	324	3.80
5	246	2.89%	208	2.43
6	166	1.93%	144	1.69
7	48	0.56%	106	1.24
8	32	0.37%	81	0.95
9	29	0.34%	64	0.75
10	23	0.27%	52	0.61
>10	100	1.17%	494	5.79
Total	8539	100.00	8,539	100.00

**Lotka's exponent (n)** by the logarithmic (least-squares) method:

$$Y=C\;x^{-n}$$

Taking logarithms on both sides gives:

Log(Y)=log(C)−nlog(X)

This is a straight-line equation:

Y=a+bX

Where:

• Y=log y
• X=log x
• a=log C
• b= −n

Table 7 provides the values of b and n as follows:

$$b=\frac{N\sum \mathrm{XY}-\sum X\sum Y}{N\sum X^{2}-{\left(\sum X\right)}^{2}}$$

**Table 7.** Computation of log x and log y.

x	Authors (y)	log _10_x	log _10_y
1	5398	0.0000	3.7322
2	1383	0.3010	3.1408
3	690	0.4771	2.8388
4	424	0.6021	2.6274
5	246	0.6990	2.3909
6	166	0.7782	2.2201
7	48	0.8451	1.6812
8	32	0.9031	1.5051
9	29	0.9542	1.4624
10	23	1.0000	1.3617

On computation:

$$\begin{array}{c} b=\frac{10\left(12.4320\right)-6.5598\left(22.9606\right)}{10\left(5.2153\right)-{\left(6.5598\right)}^{2}}\\ b=\frac{124.3200-150.6232}{52.1530-43.0310}\\ b=\frac{-26.3032}{9.1220}=-2.883 \end{array}$$

Where: N=10; **∑** logx= 6.5598; ∑ logy=22.9606; ∑ (logx)2 = 5.2153; ∑ (logx) (logy) = 12.4320

Since b= −n, the estimated Lotka exponent for the data is: n≈2.88

This indicates that the author productivity declines more steeply than the classical Lotka inverse-square law (n=2). Hence, the dataset is better represented by a modified Lotka distribution with n≈2.88 as the scatter points in Figure 3 show an approximately linear downward trend on the log–log scale. The log-log scatter plot is presented in Figure 3.

**Figure 3.**
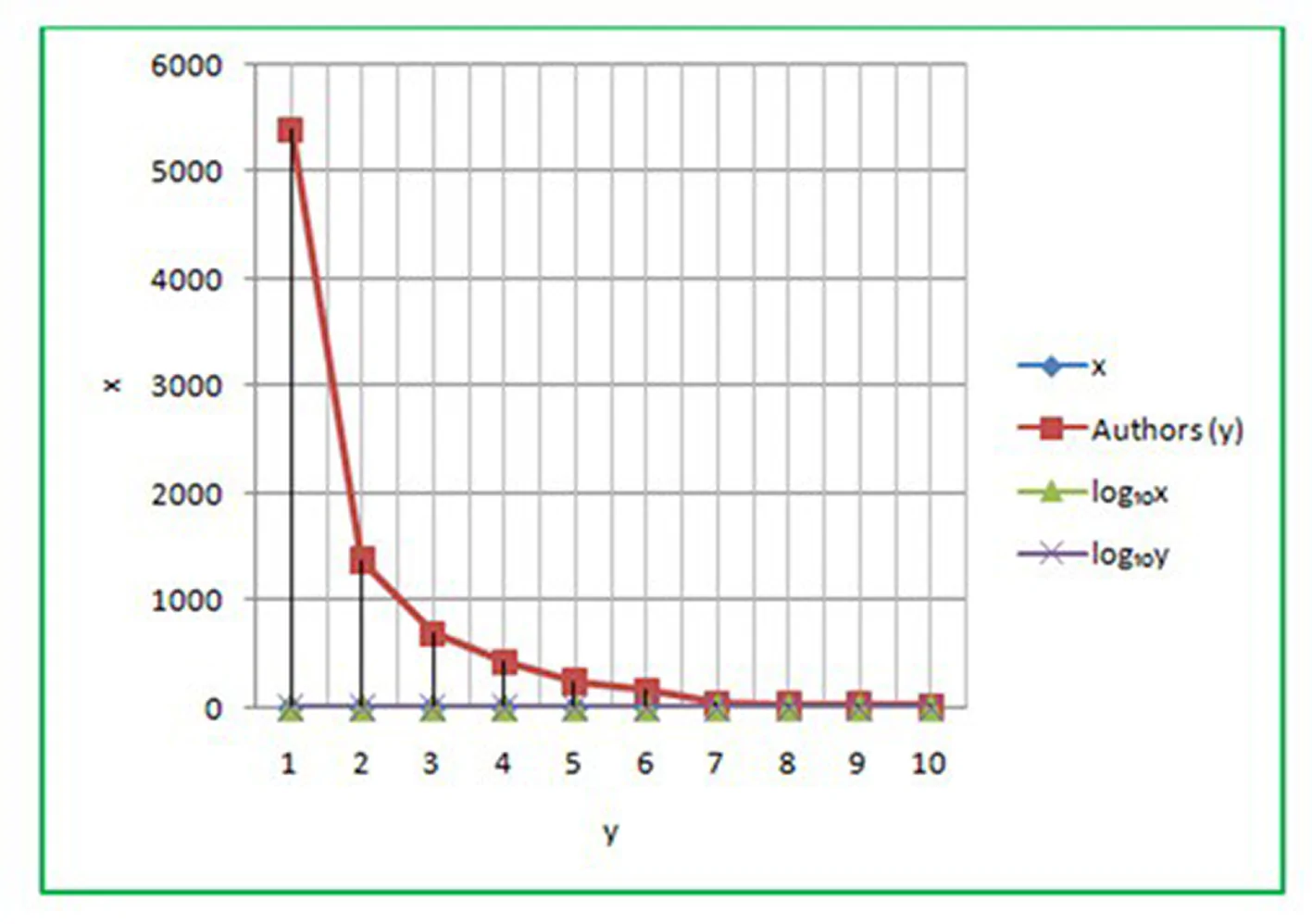
The log-log scatter plot.

Out of an initial pool of 7,136 unique author keywords, a minimum threshold of 5 occurrences was enforced to eliminate isolated topics and focus on established, repetitive institutional research trends. Under this constraint, 176 keywords met the inclusion criteria, though 4 isolated nodes with no connections to the primary component were subsequently excluded to ensure analytical continuity. Consequently, the final mapping and cluster analysis were executed on a tightly bound network of 172 items.

Table 8 reveals that the keyword “india”, which exhibits overwhelmingly dominant centrality metrics with 306 occurrences, a Total Link Strength (TLS) of 257.0, and direct connections across 98.0 distinct links. This architectural dominance signifies that the vast majority of studies published by IIMA faculty are heavily contextualized within the domestic socio-economic landscape, serving as the primary structural bridge that unifies completely disparate thematic clusters.

**Table 8.** Author Keyword co-occurrence.

Keyword	Occurrences	Total link strength	Total Link	Cluster
india	306	257	98	9
gender	34	42	21	8
covid-19	47	40	28	15
climate change	16	26	12	7
asia	12	25	13	12
bilevel optimization	20	24	8	5
sustainable development	19	23	12	7
higher education	9	22	7	14
human resource management	13	21	15	9
evolutionary algorithms	15	20	6	5
impostor phenomenon	10	19	5	14
innovation	17	19	14	6
leadership	24	19	14	13
impostor syndrome	7	18	4	14
optimization	15	18	14	5
education	15	17	14	5
emerging markets	28	17	6	2
public policy	10	17	10	12
developing countries	15	16	8	7

Notably, “covid-19” emerges as a prominent cross-cutting theme with 47.0 occurrences and a TLS of 40.0 spread over 28.0 links, highlighting how a major global health disruption heavily reshaped the institution's recent research priorities across multiple sectors simultaneously. Parallel to this, “gender” stands out as a highly dense social node with 34.0 occurrences and a TLS of 42.0 over 21.0 links, indicating a strong academic focus within the institute on identity, diversity, and equity. Furthermore, traditional business school macro-level nodes like “emerging markets” (28.0 occurrences, TLS of 17.0) and “leadership” (24.0 occurrences, TLS of 19.0) act as essential institutional gateways, effectively linking IIMA's localized regional research to broader international business frameworks and organizational theory.

From Figure 4 it is found that the surrounding topology is divided into distinct thematic domains that run both parallel and intersecting paths, reflecting IIMA's multidisciplinary profile. On the left flank of the visualization, a robust cluster emerges around computational intelligence and mathematical modeling, characterized by tightly coupled nodes such as machine learning, bi-level optimization (20.0 occurrences, TLS of 24.0), and evolutionary algorithms (15.0 occurrences). This technical wing reflects a major institutional core intensely focused on leveraging engineering, operational research, and computer science methodologies to solve complex logistical, commercial, and resource allocation problems. Conversely, the central-upper and lower-central regions are occupied by a macro-policy domain driven by keywords like sustainable development (19.0 occurrences), climate change (16.0 occurrences), and developing countries (15.0 occurrences), highlighting how the institution navigates the friction between rapid industrialization and environmental preservation. Finally, the right and lower-most edges comprise a human-centric research domain focusing on behavioral psychology, workplace dynamics, and institutional culture. This cluster connects the overarching gender hub to critical micro-level issues such as workplace bullying and job satisfaction, heavily intersecting with higher education (9.0 occurrences, TLS of 22.0) to highlight the human capital and well-being challenges accompanying the region's economic growth.

**Figure 4.**
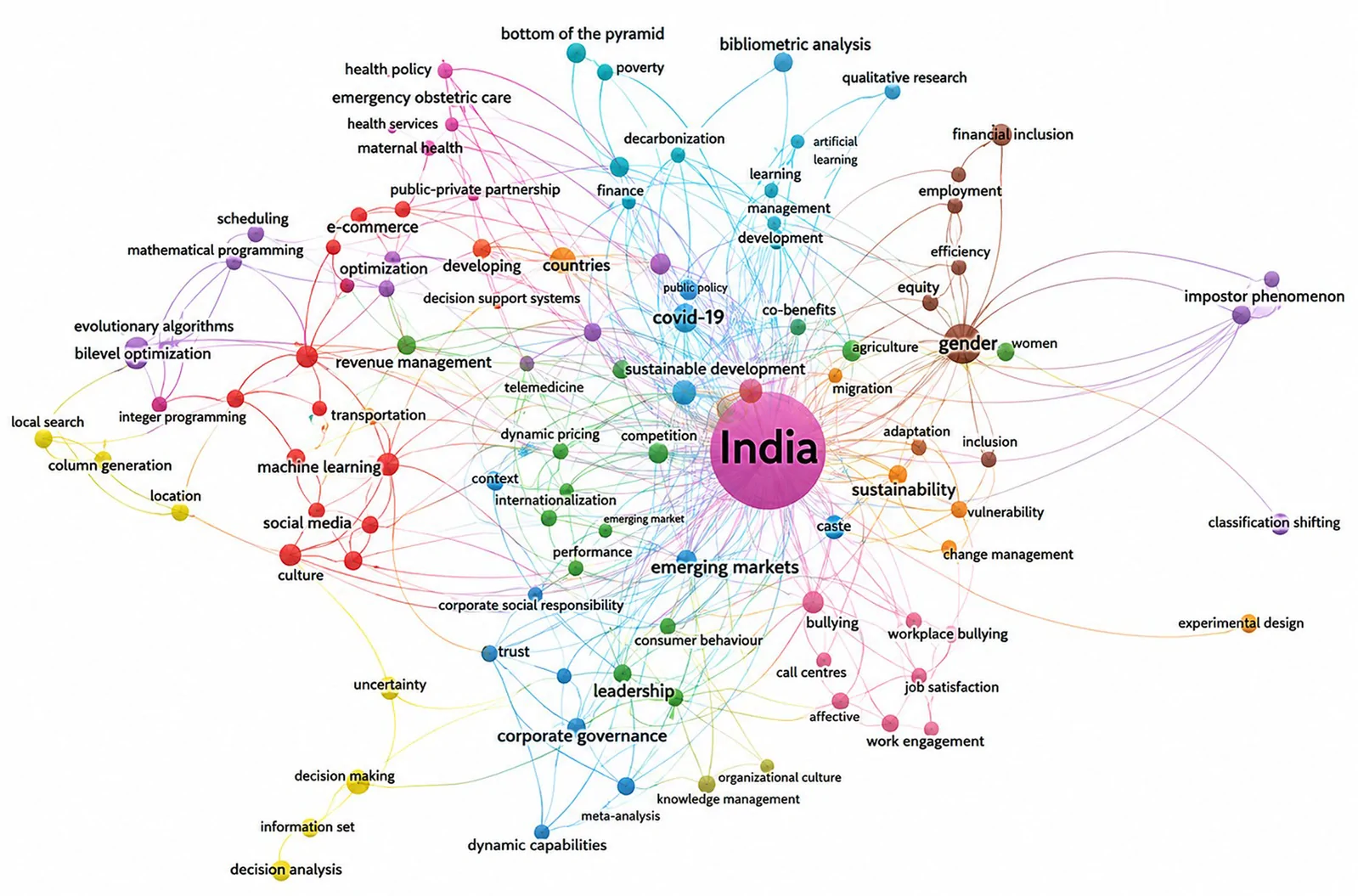
Co-Occurance keyword network analysis.

## Discussion

The present scientometric analysis provides a comprehensive overview of the research productivity and scholarly communication patterns of the Indian Institute of Management Ahmedabad (IIMA) over the period 1967–2025. The findings reveal a substantial increase in publication output over time, reflecting the institute's growing emphasis on research excellence and its expanding contribution to national and international scholarship. Although the cumulative publications from 1967–2009 account for the largest share due to the longer time span, a clear acceleration in annual research productivity is evident after 2015, with publication output reaching its peak in 2022. This upward trend is consistent with the findings of Tyagi (2024) and Singh et al. (2023), who reported a steady increase in research productivity across the Indian Institutes of Management, particularly among the older institutions.

The analysis of authorship patterns demonstrates that collaborative research has become the dominant mode of scholarly communication at IIMA. More than three-fourths (75.45%) of the publications were produced through multi-authored collaborations, resulting in a Degree of Collaboration (DC) of 0.75. The predominance of two- and three-author publications suggests that small collaborative teams are the preferred model for conducting research. The gradual increase in publications involving larger author groups further reflects the growing importance of interdisciplinary research, institutional partnerships, and international collaborations in addressing complex research problems. These findings support the widely observed global trend toward collaborative research and are consistent with earlier scientometric investigations of higher education institutions.

The analysis of prolific contributors indicates that IIMA's research output is supported by a diverse group of productive scholars rather than being concentrated among a few individuals. Although the leading authors have made substantial contributions, none accounts for more than three percent of the total publications. This balanced distribution suggests a healthy institutional research environment in which scholarly productivity is broadly shared across faculty members. Similarly, the analysis of highly cited publications highlights IIMA's significant international research impact. The presence of publications in prestigious journals such as *Science*, *The Lancet*, *IEEE Transactions on Evolutionary Computation*, and the *International Journal of Information Management* demonstrates the institute's ability to produce research of global relevance. The coexistence of highly cited methodological papers and recent publications addressing contemporary issues such as generative artificial intelligence, COVID-19, and sustainable development further illustrates the diversity and continuing relevance of IIMA's research portfolio.

The author productivity analysis revealed that the observed distribution does not conform to the classical Lotka's inverse-square law (n = 2) **.** Instead, the estimated Lotka exponent (n ≈ 2.88) indicates a steeper decline in author productivity, implying that relatively few authors contribute a large proportion of publications. Such deviations from the classical Lotka distribution have been reported in several institutional-level bibliometric studies and may reflect differences in institutional size, disciplinary composition, collaboration patterns, and faculty research specialization. Consequently, the modified Lotka distribution provides a more appropriate representation of author productivity within IIMA.

The keyword co-occurrence analysis further demonstrates the multidisciplinary nature of IIMA's research. The dominance of keywords such as India **,** COVID-19 **,** gender **,** innovation **,** leadership **,** emerging markets **,** climate change **,** sustainable development **,** among other indicates that the institute's research spans management, public policy, economics, operations research, artificial intelligence, sustainability, and social sciences. The strong interconnectedness among these thematic clusters suggests that IIMA has increasingly adopted interdisciplinary approaches to address complex societal, organizational, and technological challenges. The emergence of recent themes related to artificial intelligence, sustainability, and public health also reflects the institute's responsiveness to evolving global research priorities.

Overall, the findings indicate that IIMA has undergone a remarkable transformation from a relatively modest research-producing institution into a highly productive, collaborative, and internationally visible center of academic excellence. The continuous growth in publication output, increasing collaboration, broad distribution of scholarly contributions, and evolving research themes collectively demonstrate the institute's expanding role in advancing management education and interdisciplinary research. These findings provide valuable evidence for institutional administrators, policymakers, and research evaluators seeking to understand the dynamics of scholarly productivity and may serve as a benchmark for future comparative scientometric studies of leading management institutions in India and beyond.

## Social, academic, and managerial implications

The findings of this study demonstrate that the research output of the Indian Institute of Management Ahmedabad (IIMA) addresses several contemporary societal issues, including sustainability, climate change, public policy, gender, public health, and emerging technologies. The increasing volume of collaborative and interdisciplinary research highlights the institute's contribution to generating knowledge that can support evidence-based policymaking, sustainable development, and socio-economic progress. The identification of emerging research themes also provides valuable insights for government agencies, non-governmental organizations, and industry stakeholders in addressing complex societal challenges through research-driven solutions.

This study contributes to the scientometric literature by providing a comprehensive assessment of the research productivity, authorship characteristics, collaboration patterns, and author productivity of IIMA over nearly six decades. The application of Subramanyam's Degree of Collaboration, Lotka's Law, and VOSviewer-based keyword co-occurrence analysis demonstrates the usefulness of bibliometric techniques in evaluating institutional research performance. The findings serve as a valuable reference for researchers, librarians, and research evaluators interested in institutional benchmarking, scholarly communication, and research assessment. Furthermore, the estimated modified Lotka exponent (n ≈ 2.88) provides empirical evidence for future studies examining author productivity in management and interdisciplinary research.

The results offer important insights for institutional leaders, research administrators, and policymakers involved in research planning and evaluation. The observed growth in publication output and the high degree of collaboration indicate the effectiveness of collaborative research environments and interdisciplinary partnerships in enhancing research productivity. Identifying prolific authors, highly cited publications, and major research themes can assist the institute in formulating strategies for faculty development, research funding, mentorship, and international collaboration. Moreover, the keyword analysis highlights emerging areas of research that can guide future investments, strategic recruitment, and the establishment of interdisciplinary research centers. The findings can also support evidence-based decision-making in institutional research management, performance evaluation, and policy formulation aimed at improving the global visibility and impact of IIMA's research.

## Scope for future research

The present study is confined to publications indexed in the Scopus database and focuses primarily on publication productivity, authorship patterns, collaboration, author productivity, and keyword co-occurrence. Future research can broaden the scope by incorporating other bibliographic databases, such as Web of Science, Dimensions, and Google Scholar, to provide a more comprehensive assessment of IIMA's research output and citation impact. Comparative scientometric studies involving other leading Indian Institutes of Management, central universities, and internationally reputed business schools would also help position IIMA's research performance within national and global contexts.

Further studies may employ advanced bibliometric techniques, including co-citation analysis, bibliographic coupling, thematic evolution, citation burst analysis, and science mapping, to uncover the intellectual structure and evolution of research domains. Social network analysis of institutional, national, and international collaborations could provide deeper insights into knowledge-sharing networks and research partnerships. Additionally, future research may examine the impact of open-access publishing, research funding, international collaboration, and interdisciplinary research on citation performance and research visibility. Longitudinal studies investigating the influence of emerging themes such as artificial intelligence, sustainability, digital transformation, and public policy on IIMA's research agenda would further enhance the understanding of the institute's evolving scholarly contributions.

## Conclusion

The scientometric analysis of publications produced by the Indian Institute of Management Ahmedabad (IIMA) during the period 1967–2025 reveals a steady and significant growth in the institute's research output, with a marked increase in publications during the last decade. The findings indicate that IIMA has established a strong research culture characterized by increasing scholarly productivity and expanding academic contributions across diverse disciplines. The authorship analysis demonstrates that collaborative research is the predominant mode of scholarly communication, with nearly three-fourths of the publications involving multiple authors and a Degree of Collaboration of 0.754, reflecting the growing importance of teamwork, interdisciplinary research, and institutional partnerships. The analysis of prolific authors and highly cited publications further highlights the presence of several influential researchers whose contributions have achieved considerable international recognition and citation impact. Examination of author productivity using Lotka's Law shows that the observed distribution does not conform to the classical inverse-square law (n = 2). The estimated Lotka exponent (n ≈ 2.88) indicates a steeper decline in author productivity, suggesting that the research output of IIMA is better represented by a modified Lotka distribution. Overall, the study demonstrates that IIMA has evolved into a highly productive and collaborative research institution with increasing global visibility, making substantial contributions to scientific knowledge and scholarly communication.

## Data Availability

No data associated with this article. The data for this article consists of bibliographic references, which are included in the references section.
