# Supplementary material for: Scholarly Communication and Authorship Pattern of Indian Institute of Management Ahmedabad: a Scientometric Analysis
Source: F1000Res. 2026 Aug 20;15:1402. [Version 1] doi: 10.12688/f1000research.188052.1 (PMC13755622; doi:10.12688/f1000research.188052.1)
Supplement: Peer review history

# Reviewer report: Reviewer response for version 1

**DOI:** 10.5256/f1000research.207536.r538922

**Published:** 2026-11-11

**Authors:** Anita Helen

**Affiliations:** 1 Department of Library and Information Science, National University, Gazipur, Bangladesh

I have gone through the manuscript titled “Scholarly Communication and Authorship Pattern of Indian Institute of Management Ahmedabad: A Scientometric Analysis” and recommend that it can be accepted. The study analyses 2,981 Scopus-indexed publications of IIMA from 1967 to 2025. It looks at publication growth, authorship patterns, author productivity, collaboration and keyword co-occurrence. The authors use standard bibliometric indicators, Lotka’s Law and VOSviewer for this purpose. The topic is relevant, and the objectives are clearly stated. The paper is well organised, and it cites the current literature properly. The methodology suits the aims of the study, and the data source, study period and analytical tools are described well enough for others to replicate the work. The analysis and its interpretation are appropriate. The Degree of Collaboration is 0.75, which shows a strong preference for collaborative research. The modified Lotka exponent is about 2.88. The conclusions are supported by the findings. The discussion links the results with earlier studies, and the implications and future research directions add value to the paper. The study will be useful for researchers, librarians, academic administrators and policymakers. I would only suggest that the authors share the exported Scopus dataset and supplementary files, which would further strengthen reproducibility. A final check of the numbers and the language before indexing would also help. Overall, the manuscript meets the required scholarly standard, and I recommend it for indexing.

Is the work clearly and accurately presented and does it cite the current literature?

Yes

If applicable, is the statistical analysis and its interpretation appropriate?

Yes

Are all the source data underlying the results available to ensure full reproducibility?

Yes

Is the study design appropriate and is the work technically sound?

Yes

Are the conclusions drawn adequately supported by the results?

Yes

Are sufficient details of methods and analysis provided to allow replication by others?

Yes

Reviewer Expertise:

NA

I confirm that I have read this submission and believe that I have an appropriate level of expertise to confirm that it is of an acceptable scientific standard.

# Reviewer report: Reviewer response for version 1

**DOI:** 10.5256/f1000research.207536.r538925

**Published:** 2026-11-10

**Authors:** Lakshminarasimhappa M C

**Affiliations:** 1 Bangalore University, Bengaluru, Karnataka, India; 2 Central Library, Amity University, Hyderabad, Telangana, India

1. Is the work clearly and accurately presented, and does it cite the current literature? Partly

The required rectifications must be carried out:

- Tables 1 and 4 give different yearly totals, though both sum to 2,981.
- Vohra N's share is 2.71% in Table 2, but 51/2,981 is 1.71%.
- DC is reported in four ways: 0.75, 75.44%, 75.45% and 0.754.
- Figure 3 is not a log-log plot, and the VOSviewer version is left as "X.X".
- Objectives and research questions overlap.
- Many cited studies come from unrelated fields, and the closest IIM studies are not used for benchmarking.
- Foundational sources on Lotka's Law and VOSviewer are missing.

The authors are advised to reconcile Tables 1, 4, and Figure 1; correct the Vohra N percentage; report DC consistently; replace Figure 3; state the software version; and fix the data availability statement. Authors should merge overlapping objectives, cite the missing methodological literature, and compare IIMA with Singh et al. 2023, Tyagi 2024 and Sahoo et al. 2017.

 2. Is the study design appropriate and is the work technically sound? Partly

 The required rectifications must be carried out:

- There is no comparator or normalisation, so "highly productive" has no benchmark.
- The paper promises "scholarly communication" but analyses no journals, document types or open access.
- Pooling 1967–2009 into one row hides the long-term trend.
- Interdisciplinary, institutional and international collaboration are claimed but never measured.
- Lotka's Law is applied to all 8,539 co-authors, most of whom are probably external to IIMA.

Authors must state whose authors are counted (preferably repeat the analysis for IIMA authors only), and drop or substantiate the collaboration claims. Authors should add a comparator such as IIM Bangalore or Calcutta, show decade-level trends, analyse co-author countries and subject areas, and add journal analysis or retitle the paper.

 3. Are sufficient details of methods and analysis provided to allow replication by others? No

 The required rectifications must be carried out:

- The Scopus query, affiliation variants and retrieval date are missing.
- Document types and languages are not stated.
- Full vs. fractional counting and author disambiguation are not described.
- It is unclear how the Lotka range (x = 1–10) and the ">10" group were handled, or how expected values were calculated.
- VOSviewer parameters and keyword cleaning are not given.

Authors must report all of the above. Authors are required to add a flow diagram showing record counts from retrieval to the final dataset.

 4. If applicable, is the statistical analysis and its interpretation appropriate? No

 The required rectifications must be carried out:

- Σ(log x · log y) recomputes to about 12.82, not 12.43, which gives n ≈ 2.46 rather than 2.88.
- There is no K–S test or other goodness-of-fit test.
- R², standard error and confidence interval are missing. My approximate CI of 2.0–2.9 means the departure from n = 2 is less firm than claimed.
- Unweighted least squares on ten points is a weak estimator.
- The interpretation is reversed: a steeper exponent means fewer prolific authors, not "few authors contribute most".
- No trend statistics support "increasing collaboration". My rough decade values are DC ≈ 0.50 (1967–79) and ≈ 0.87 (2020–25).

Authors must recompute the exponent, report R², SE, CI, and a K–S test, correct the interpretation, and update the abstract, discussion, and conclusion. Authors should use maximum-likelihood or Pao's method, show decade-level DC, add growth rates, and run a sensitivity check excluding hyper-authored papers.

 5. Are all the source data underlying the results available to ensure full reproducibility? No

 Problems

- The statement says "No data" and then claims the data are the references.
- The reference list is the cited literature, not the 2,981 records.

Authors must deposit the EIDs, query and retrieval date in a public repository; deposit the author-level data behind Table 6, the calculation files, and the VOSviewer files; and rewrite the statement. Authors should provide machine-readable versions of Tables 1 and 4.

 6. Are the conclusions drawn adequately supported by the results? Partly

 Supported

- Output has risen since about 2015.
- Most papers are multi-authored.
- The distribution probably departs from n = 2.

Not supported

- "Internationally recognised" and "highly productive": there is no benchmark or country data.
- "Continuous growth": output fell after 2022.
- "Vast majority India-contextualised": the keyword appears in only about 10% of records.
- "Healthy" environment, "influential researchers", and causal or policy claims.

Authors must reword or substantiate the evaluative claims, discuss the post-2022 decline, and correct the India-keyword statement.

 Authors should state limitations clearly (Scopus-only coverage, affiliation matching, author ambiguity, full counting) and remove or label causal and policy claims as speculative.

 Final recommendation

 Approved with Major Revisions: The design is acceptable, and the problems are fixable. If the authors cannot reconcile Tables 1 and 4, the recommendation should become Not Approved.

 Authors must address the points below

1. Recompute the Lotka exponent and add R², SE, CI and a K–S test.
2. Correct the Lotka interpretation.
3. Reconcile Tables 1, 4 and Figure 1, and fix the Vohra N percentage and DC reporting.
4. Replace Figure 3 and fix the software version.
5. State which authors Lotka's Law covers.
6. Report the full search strategy and counting methods.
7. Document VOSviewer settings and keyword coverage.
8. Fix the data statement and deposit the data.
9. Remove or support the unsupported claims.
10. Add limitations.

Is the work clearly and accurately presented and does it cite the current literature?

Partly

If applicable, is the statistical analysis and its interpretation appropriate?

No

Are all the source data underlying the results available to ensure full reproducibility?

No

Is the study design appropriate and is the work technically sound?

Partly

Are the conclusions drawn adequately supported by the results?

Partly

Are sufficient details of methods and analysis provided to allow replication by others?

No

Reviewer Expertise:

Metric Studies; Advanced scientometrics; Bibliometrics; Digital Archives; Digital Library

I confirm that I have read this submission and believe that I have an appropriate level of expertise to confirm that it is of an acceptable scientific standard, however I have significant reservations, as outlined above.

# Reviewer report: Reviewer response for version 1

**DOI:** 10.5256/f1000research.207536.r513690

**Published:** 2026-08-28

**Authors:** Shivam Pachauri

**Affiliations:** 1 Library, Sanskriti University (Ringgold ID: 602220), Mathura, Uttar Pradesh, India; 2 Mangalayatan University, Aligarh, Uttar Pradesh, India

This article is well-structured and clear, and it is deeply grounded in the fields of scientometrics and management studies. The background highlights the significance of the study by emphasizing key aspects such as globalization, digital transformation, sustainability, and technological innovation. The methodology is described in detail, including comprehensive information regarding the datasets used. The findings are concisely presented, offering interesting insights into publication scope, collaborative research, and author output. The conclusion successfully encapsulates the study's primary contributions, highlighting the institution's evolution into an efficient, collaborative, and globally recognized entity. Overall, the article is methodologically sound, informative, and professionally crafted, effectively outlining the research objectives, methodology, key findings, and significance. The manuscript demonstrates strong potential for enhancing scientific inquiry and assessing the effectiveness of institutional scientific research.

Is the work clearly and accurately presented and does it cite the current literature?

Yes

If applicable, is the statistical analysis and its interpretation appropriate?

Yes

Are all the source data underlying the results available to ensure full reproducibility?

Yes

Is the study design appropriate and is the work technically sound?

Yes

Are the conclusions drawn adequately supported by the results?

Yes

Are sufficient details of methods and analysis provided to allow replication by others?

Yes

Reviewer Expertise:

Scientometric , Bibliometric, Digital Literacy, Artificial Intelligence in Libraries

I confirm that I have read this submission and believe that I have an appropriate level of expertise to confirm that it is of an acceptable scientific standard.

# Response

**Published:** 2026-08-28

**Authors:** Dr. Dillip K Swain

**Affiliations:** Library & Information Science, Kalinga Institute of Industrial Technology, Bhubaneswar, Odisha, India

We would like to express our deepest gratitude and sincere appreciation to the esteemed reviewer for the meticulous evaluation of our manuscript and for the valuable time and expertise devoted to its review. We are truly honored by the reviewer’s positive assessment and gracious acceptance of the manuscript in its present form. The reviewer’s kind appreciation is a great encouragement to us and is deeply valued.
